# When Endoscopic Sedation is Not an Option: Insights From a Multicenter on‐site Survey on Tolerance for Japanese Gastric Cancer Screening

**DOI:** 10.1002/deo2.70179

**Published:** 2025-08-11

**Authors:** Kazuya Inoki, Toshiya Chiba, Kazuhiro Miura, Teppei Tagawa, Yutaka Aoyagi, Yoshiaki Takeuchi, Naoya Moriyama, Kensuke Kuraoka, Tadashi Ohara, Yuko Haruhara, Akira Mizuki, Jun Satio, Yoshimi Hagisawa, Naomi Ono, Noboru Takayama, Takeo Onishi, Akira Mishima, Norihiro Suzuki, Kazuo Kikuchi, Atsushi Katagiri, Fuyuhiko Yamamura, Sachiko Ohde, Eisuke Inoue, Takahisa Matsuda, Hitoshi Yoshida

**Affiliations:** ^1^ Department of Medicine Division of Gastroenterology Showa Medical University School of Medicine Tokyo Japan; ^2^ Chiba Medical Dental Clinic Tokyo Japan; ^3^ Miura Clinic Tokyo Japan; ^4^ Tagawa Clinic Yokohama Japan; ^5^ Aoyaghi Clinic Tokyo Japan; ^6^ Takeuchi Gastrointestinal Medicine Clinic Tokyo Japan; ^7^ Moriyama Clinic Tokyo Japan; ^8^ Osaki Gastroenterology Clinic Tokyo Japan; ^9^ Department of Aging Research and Geriatric Medicine Institute of Development Aging and Cancer Tohoku University Sendai Japan; ^10^ Hatanodai Hospital Tokyo Japan; ^11^ Tokyo Sea Fort Square Clinic Tokyo Japan; ^12^ Saito Family Clinic Tokyo Japan; ^13^ Ohi Chuou Hospital Tokyo Japan; ^14^ Minoyama Hospital Kyoto Japan; ^15^ Takayama Clinic Tokyo Japan; ^16^ Onishi Clinic Tokyo Japan; ^17^ Mishima Internal Medicine Tokyo Japan; ^18^ Endoscopy Center Showa Medical University Hospital Tokyo Japan; ^19^ Graduate School of Public Health St. Luke's International University Tokyo Japan; ^20^ Showa Medical University Research Administration Center Tokyo Japan; ^21^ Division of Gastroenterology and Hepatology Toho University Omori Medical Center Tokyo Japan

**Keywords:** digestive system, endoscopy, moderate sedation, patient acceptance of health care, stomach neoplasms

## Abstract

**Objectives:**

Esophagogastroduodenoscopy (EGD) is widely used for gastric cancer (GC) screening in Japan; however, sedation during EGD is not recommended. We aimed to assess the tolerability of unsedated EGD (USEGD) in the Japanese population.

**Methods:**

Participants who underwent GC screening in Japanese urban areas between July 2021 and December 2022 were included in this study. We conducted a real‐time questionnaire survey on USEGD invasiveness in 16 clinics and hospitals. Participants completed a self‐report questionnaire, including a six‐point face scale, immediately after undergoing USEGD with an ultrathin endoscope for GC screening, and were placed into the tolerable (T group) or intolerable groups (I group) based on the scores 1–3 and 4–6 on the face scale, respectively.

**Results:**

The 1021 participants (median age, 59 years; interquartile range, 59–74 years) consisted of 561 men and 437 women, while 23 preferred not to answer. Of the 777 participants who underwent USEGD using an ultrathin endoscope, 135 (17.4%) were categorized into the intolerable (I) group based on severe distress, and 642 (82.6%) into the tolerable (T) group. Multiple logistic regression analysis revealed that older age (odds ratio [OR]: 0.974; *p* = 0.008) and prior USEGD experience (OR: 0.527; *p* = 0.006) were associated with higher tolerability. Conversely, females (OR: 2.498; *p* < 0.001) and first‐time EGD experience (OR: 2.202; *p* = 0.003) were associated with lower tolerability.

**Conclusions:**

USEGD was generally well‐tolerated; however, some participants found it intolerable, even with transnasal endoscopy. Supportive measures for these individuals are essential for sustaining effective screening programs.

## 1 Introduction

Gastric cancer (GC) has been a primary focus of cancer screening and prevention in Japan. It is a major public health concern, and despite declining mortality trends in both sexes, it ranks as the third and fifth leading cause of death among men and women, respectively [1, 2] Based on robust evidence that GC screening reduces mortality, a population‐based screening program has been implemented in Japan since the 1960s [3]. Traditionally, the upper gastrointestinal series has been the primary method for GC screening in Japan, with esophagogastroduodenoscopy (EGD) used only for follow‐up diagnostic examination of abnormal upper gastrointestinal series findings. However, EGD has recently been approved as a primary screening method [3, 4].

Minimizing the risk of adverse events is critical when implementing cancer‐screening programs for healthy individuals. The incidence of adverse events is low; however, such events associated with sedation during EGD were reported in a nationwide Japanese survey conducted by the Japanese Gastroenterological Endoscopy Society [5]. To eliminate these risks, unsedated EGD (USEGD) is recommended for GC screening in Japan by the Japanese Society of Gastrointestinal Cancer Screening [3]. However, in clinical settings, sedation for EGD is often preferred and demanded by patients due to the discomfort associated with USEGD. A meta‐analysis demonstrated that sedation increases patient satisfaction and willingness to repeat EGD procedures [6]. Furthermore, a randomized controlled trial showed that sedated EGD improved both patient and GI endoscopists’ satisfaction [7]. Transnasal EGD using an ultrathin endoscope is a common and more relaxing procedure than oral endoscopy; however, some patients still struggle to tolerate it due to factors including a narrow nasal cavity or severe throat reflex [8].

Currently, the Japanese GC screening guidelines lack specific policies for examinees unable to tolerate USEGD. This raises concerns that fear of USEGD may discourage participation in GC screening programs. The tolerability of USEGD in real‐world GC screening settings and the factors influencing intolerance remain unclear.

In the present study, we conducted an on‐site questionnaire survey to evaluate the tolerance of examinees undergoing GC screening using USEGD in a Japanese real‐world setting.

## 2 Methods

### 2.1 Study Design and Participants

We conducted an on‐site written questionnaire survey at 16 clinics and hospitals in the Shinagawa Ward, Tokyo, and the Seya Ward, Kanagawa Prefecture. The study was approved by the Institutional Review Board of Showa University Hospital (M3475). Participants who underwent population‐based GC screening between July 2021 and December 2022 were included in this study and were asked to complete the questionnaire immediately after undergoing EGD as part of GC screening at each institution. Individuals who underwent screening but did not consent to the questionnaire survey were excluded from the analysis.

### 2.2 Questionnaire and Data Collection

Completed questionnaires were collected via mail from the respective institutions. The first item of the questionnaire sought consent for participation, and only those who provided consent answered the remaining questions, which included the following:

1. How many times have you undergone EGD?
2. Have you ever undergone a sedated EGD?
3. How do you feel about today's EGD?
4. Do you think you can undergo USEGD again?
5. What was your impression of the examination duration?
6. How did you feel after the EGD?
7. What do you think about sedation during an EGD?
8. Do you want to undergo a sedated EGD despite the risk of adverse events associated with EGD?
9. Do you want to undergo a sedated EGD despite the restrictions of EGD? and,
10. Do you think you can undergo USEGD every few years?

Medical staff at each institution filled out items regarding the insertion route, biopsy presence or absence, and examination time. Questions 3–6 used a 6‐grade face scale, with 1 corresponding to “most comfortable” and 6 to “most uncomfortable.” A detailed description of the questionnaire is provided in Table S1.

### 2.3 Outcome Measures

We assessed the proportion of participants who experienced severe distress during USEGD. To further investigate USEGD tolerability and the factors associated with severe distress during the procedure, participants who underwent USEGD with an ultrathin scope were divided into two groups: tolerable (T group) and intolerable (I group), with both groups including participants who scored 1–3 and 4–6, respectively, on the face scale for question (4). Participants with missing data and those who underwent USEGD with a conventional scope (65 participants) were excluded from the study, as it is well‐recognized that distress levels differ significantly between conventional and ultrathin endoscopes.

### 2.4 Standards for Physician Qualifications in Performing EGD for Population‐based GC Screening in Japan

In the population‐based GC screening program in Japan, qualification requirements for physicians who perform upper gastrointestinal endoscopy are defined by the Japanese Society of Gastrointestinal Cancer Screening. Eligible physicians must meet at least one of the following three criteria:

1. be certified as a Board‐Certified Member or General Board‐Certified Member of the Japanese Society of Gastrointestinal Cancer Screening, a Board‐Certified Fellow of the Japan Gastroenterological Endoscopy Society, or a Board‐Certified Fellow of the Japanese Society of Gastroenterology;
2. perform approximately 100 or more upper gastrointestinal endoscopic procedures per year; or
3. be recognized by the Endoscopic Screening Steering Committee as having equivalent experience and competence to those meeting criteria (1) or (2), based on a formal eligibility assessment.

In addition to satisfying one of the above criteria, all endoscopists must undergo a competency evaluation by the Screening Steering Committee and are required to attend designated training sessions or case review meetings at least once annually.

### 2.5 Endoscope Selection and Insertion Practices across Institutions

The endoscopes used at each institution were as follows:

Conventional‐diameter endoscopes: Olympus GIF‐H290; Fujifilm EG‐760Z and EG‐L600ZW7.

Ultrathin endoscopes: Olympus GIF‐1200N, GIF‐H290N, GIF‐H190N, and GIF‐XP290N; Fujifilm EG‐L580NW7, EG‐840N, and EG‐6400N.

The choice of insertion route varied across the participating institutions. In addition, there are no standardized regulations on endoscope types in population‐based GC screening programs. In five facilities, both ultrathin endoscopes and conventional‐diameter endoscopes were employed due to limitations in scope reprocessing capacity. At the other institutions, transnasal endoscopy with ultrathin scopes was routinely performed. However, in all institutions using ultrathin scopes, the oral route was chosen instead when patients expressed a preference or when nasal insertion was anatomically difficult.

### 2.6 Statistical Analysis

The target sample size was initially set at 400 participants per group (first‐time EGD vs. prior EGD experience). The required sample size was estimated based on a 95% confidence level and a margin of error of ±5%. Under the conservative assumption that the true population proportion is 50%—which yields the maximum variability and thus the largest required sample size—a minimum of 385 participants is necessary when assuming an infinite population. While the required sample size may be slightly adjusted when the total population is small, the difference becomes negligible once the population exceeds 10,000. Therefore, a sample size of approximately 400 is generally considered sufficient for surveys targeting large populations, including participants of GC screening. After accounting for an expected survey response rate of 80%, the final target sample size was adjusted to 1,000 participants (500 per group). However, upon study completion, it became evident that the proportion of first‐time EGD examinees was lower than anticipated, making it difficult to achieve the target number of 400 patients. Considering the burden on the participating institutions, the study concluded with the originally planned number of 1000 participants.

Continuous variables were expressed as median with interquartile range [IQR] and full range, and compared using the Mann–Whitney U test. Categorical variables were expressed as numbers and percentages and compared using the chi‐squared test. To identify factors associated with intolerance to USEGD, we performed multivariate logistic regression using intolerance (1 = intolerant, 0 = tolerant) as the dependent variable. Independent variables included age, sex, EGD history, prior sedation, insertion route, and examination time. Adjusted odds ratios (aORs) with 95% confidence intervals were calculated. A *p*‐value < 0.05 was considered statistically significant. All analyses were conducted using R software (v4.3.1; R Foundation, Vienna, Austria). Although no standard exam time exists for GC screening with an ultrathin scope, we used 7 min as the cutoff, based on evidence that endoscopists with average EGD times over 7 min identified significantly more high‐risk gastric lesions than those with shorter durations [9].

## 3 Results

Of the 1063 individuals approached, 1021 (95.1%) provided informed consent and were included in the analysis; the remaining 42 were excluded due to a lack of consent. Table 1 summarizes the participant characteristics and USEGD details. The median age was 59 years (interquartile range: 59–74; range: 40–93 years). The sex distribution included 561 men, 437 women, and 23 participants who preferred not to answer. Regarding EGD experience, 134 participants were first‐time users, 680 had undergone EGD two to five times, and 204 had undergone EGD more than five times; data were missing for three participants. For the USEGD experience, 188 participants reported prior experience, while 779 had no prior experience, of which 113 were USEGD first‐timers, and 34 provided no information. The endoscope insertion routes included transnasal with an ultrathin scope (594 participants), transoral with an ultrathin scope (232 participants), and transoral with a conventional endoscope (65 participants); however, 130 participants did not provide this information. Biopsies were performed in approximately 5% of participants. The median procedure time was 7 min.

**TABLE 1:** Characteristics of participants and unsedated esophagogastroduodenoscopy.

*n* = 1021	
Age, years, median (IQR, range)	59 (59–74, 40–93)
Sex, *n* (%)	
Men	561 (54.9)
Women	437 (42.8)
No answer	23 (2.3)
EGD experience, *n* (%)	
First time	134 (13.1)
Several times (2–5 times)	680 (66.6)
Many times (>5 times)	204 (20.0)
No answer	3 (0.3)
EGD sedation experience, *n* (%)	
Yes	188 (18.4)
No (First‐time)	113 (11.1)
No (Not first‐time)	686 (67.1)
No answer	34 (3.3)
EGD insertion route, *n* (%)	
Nasal	594 (58.2)
Oral with a small‐caliber endoscope	232 (22.7)
Oral with a conventional endoscope	65 (6.4)
No answer	130 (12.7)
Biopsy during EGD, *n* (%)	
Yes	53 (5.2)
No	829 (81.2)
No answer	139 (13.6)
Procedure time, *n* (%)	
≤7 min	513 (50.2)
>7 min	396 (38.8)
No answer	112 (11.0)

Abbreviations: EGD, esophagogastroduodenoscopy; IQR, interquartile range.

**FIGURE 1:**
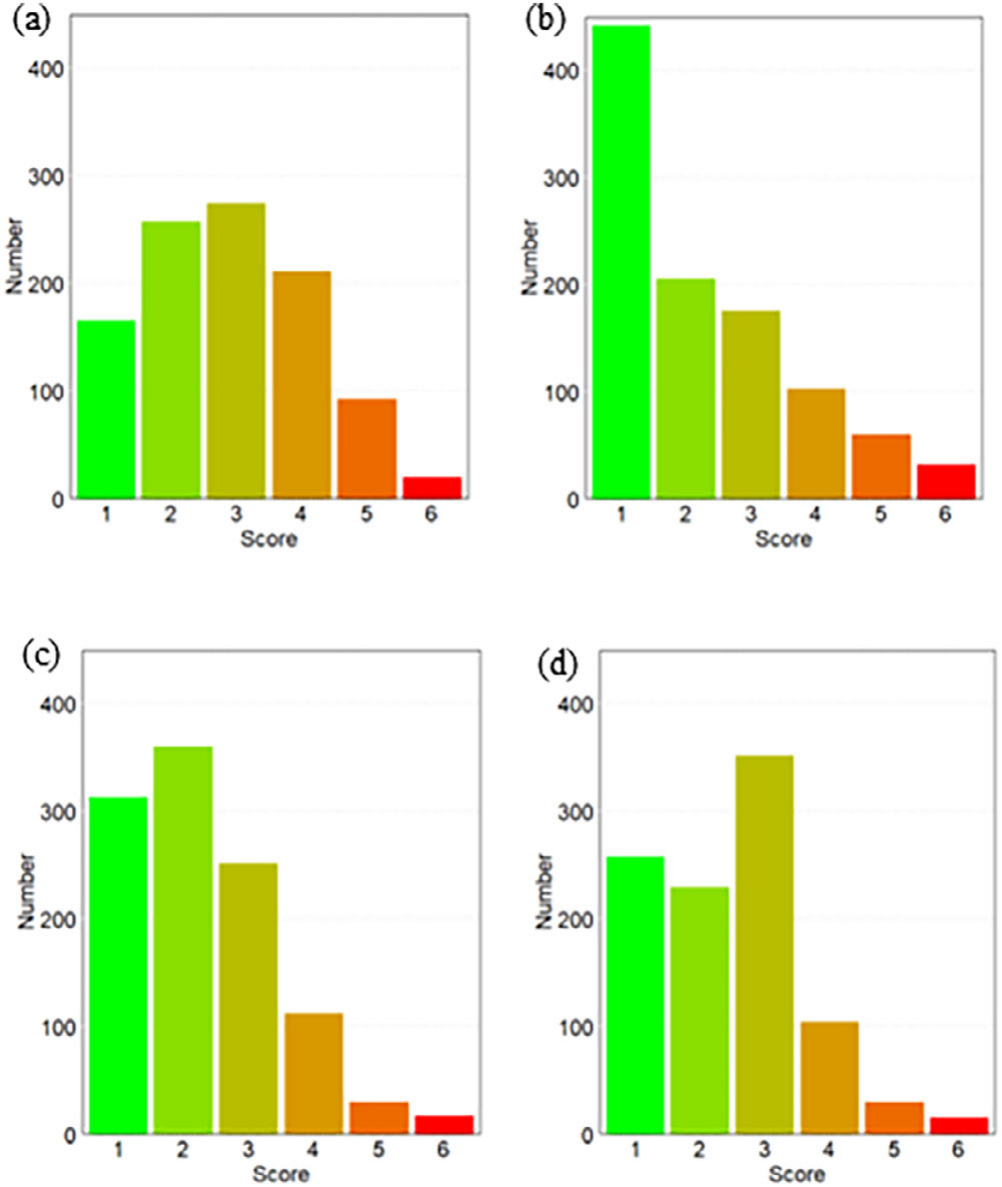
Bar chart showing participants’ feedback on their unsedated esophagogastroduodenoscopy (USEGD) experience. The figure summarizes responses to the following questions: (a) How did you feel about today's EGD? (b) Do you think you could undergo USEGD again? (c) What is your impression of the examination duration? (d) How did you feel after the EGD?

Figure 1 summarizes the responses regarding distress during USEGD [Questions (3)—(6)], demonstrating that the number of participants who could tolerate USEGD exceeded those who found it intolerable. The questions and answers related to sedation preferences, risks, and restrictions are summarized in Table 2. Regarding sedation preference, 4.3% and 35.0% of participants responded with “Definitely yes” and “Yes, if possible,” respectively. After excluding participants who had undergone USEGD using the conventional scope and those with missing data, we included 777 participants for analysis. Among these, 135 (17.4%; 95% confidence interval [CI]: 14.8%–20.1%) experienced severe distress during the USEGD. Table 3 shows the characteristics of the participants who underwent USEGD using a thin scope. The participants in the T group were older than those in the I group (67.5 years old vs. 64.2 years old). The proportion of women in the I group was higher than that in the T group (54.2% vs. 75.6%). Furthermore, the proportion of participants with prior experience of EGD and no prior experience of sedation during EGD was higher in the T group than in the I group (88.2% vs. 79.3% and 82.1% vs. 73.3%, respectively). Among participants undergoing their first EGD with an ultrathin scope, the estimated proportion who were intolerant was 26.9% (95% CI: 18.9%–36.4%). Multiple logistic regression analysis revealed that advanced age (aOR, 0.974; *p* = 0.008) and no prior experience of sedation during EGD (aOR, 0.527; *p* = 0.006) were associated with a higher tolerability of USEGD. Conversely, being female (aOR, 2.498; *p* < 0.001) and undergoing EGD for the first time (aOR, 2.202; *p* = 0.003) were associated with higher intolerability of USEGD, as shown in Table 4.

**TABLE 2:** Questions and answers regarding sedation preference, risk, and duration of esophagogastroduodenoscopy.

Questions	*n* (%)
Would you require sedation for an EGD procedure?	
Definitely yes	44 (4.3)
Yes, if possible	357 (35.0)
No	580 (56.8)
No answer	40 (3.9)
The risk of death due to sedative EGD is reported as 0.000024%.	
Based on the risk, do you need sedation during EGD?	
Yes	242 (23.7)
No	666 (65.2)
Others	29 (2.8)
No answer	84 (8.2)
Recovery time after examination and restriction of driving or work is necessary if you choose sedation. Based on the information, do you need sedation during EGD?	
Yes	213 (20.9)
No	711 (69.6)
Others	21 (2.1)
No answer	76 (7.4)
Do you think that you can undergo unsedated EGD every few years?	
Yes	864 (84.6)
No	105 (10.3)
No answer	52 (5.1)

Abbreviation: EGD, esophagogastroduodenoscopy.

**TABLE 3:** Characteristics of participants who underwent unsedated esophagogastroduodenoscopy using ultrathin scope.

	T group (*n* = 642)	I group (*n* = 135)	*p*‐Value
Age, mean (SD)	67.5 (9.7)	64.2 (10.4)	<0.001
Sex, (%)			
Men	294 (45.8)	33 (24.4)	<0.001
Women	348 (54.2)	102 (75.6)	
EGD experience, *n* (%)			
Yes	566 (88.2)	107 (79.3)	0.009
No	76 (11.8)	28 (20.7)	
EGD sedation experience, *n* (%)			
Yes	115 (17.9)	36 (26.7)	0.027
No	527 (82.1)	99 (73.3)	
EGD insertion route, *n* (%)			
Nasal	460 (71.7)	98 (72.6)	0.908
Oral	182 (28.3)	37 (27.4)	
Procedure time, *n* (%)			
≤7 min	361 (56.2)	81 (60.0)	0.479
>7 min	281 (43.8)	54 (40.0)	

Abbreviations: I, intolerable group; SD, standard deviation; T, tolerable group.

**TABLE 4:** Results of logistic regression analysis to investigate the factors associated with tolerability and intolerability of unsedated esophagogastroduodenoscopy.

	**Adjusted ORs**	**95% CI**	***p*‐Value**
Age	0.974	0.955–0.993	0.008
Sex			
Men	Reference		
Women	2.498	1.642–3.883	<0.001
EGD experience			
Yes	Reference		
No	2.202	1.299–3.673	0.003
EGD sedation experience			
Yes	Reference		
No	0.527	0.334–0.840	0.006
Insertion route			
Nasal	Reference		
Oral	1.002	0.647–1.529	0.991
Procedure time			
≤7 min	Reference		
>7 min	0.84	0.566–1.240	0.384

Abbreviations: CI, confidence interval; EGD, esophagogastroduodenoscopy; ORs, odds ratio; USEGD, unsedated esophagogastroduodenoscopy.

## 4 Discussion

According to the Shinagawa City Cancer Control Promotion Plan, only 38.3% of residents underwent GC screening in 2023, indicating suboptimal participation. To investigate potential barriers, we conducted a real‐time, on‐site questionnaire survey assessing tolerance to unsedated USEGD. The findings revealed diverse participant preferences: while most found USEGD tolerable, some rejected it even via the transnasal route. Younger women and first‐time examinees were more likely to report significant distress.

In Japan, population‐based screening targets gastric, colorectal, lung, uterine, cervical, and breast cancers. Safety is a priority in such programs because they involve large numbers of healthy individuals without target diseases. Sedation during endoscopy carries the risk of adverse events, including respiratory depression and hypoxemia [10], necessitating careful monitoring by healthcare providers [11, 12, 13]. Extended recovery time and work restrictions are further disadvantages of sedation during endoscopy [14], which can be considered as indirect sedation costs. Sedation is not recommended for EGD in GC screening in Japan, due to safety concerns and time constraints for both participants and healthcare providers. Therefore, the transnasal route has become essential for USEGD procedures. Several studies have concluded that transnasal EGD offers higher patient acceptance and preference than transoral EGD [8, 15, 16, 17, 18, 19]. Low diagnostic sensitivity remains the primary concern with ultrathin endoscopy [20, 21]; however, recent advancements in ultrathin endoscopes and endoscopic systems, including new image‐enhanced endoscopy techniques, address this issue [22, 23]. Although ultrathin endoscopy is a logical approach, many participants still experienced considerable stress during transnasal USEGD, indicating that it does not fully resolve real‐world challenges.

The results of this study provide important insights into the risk factors of stress during USEGD. Young women may report pain more frequently than older men, a finding that aligns with physicians’ observations in daily clinical practice. Furthermore, undergoing EGD for the first time or having prior experience with sedative EGD were identified as significant risk factors. These findings highlight a critical concern that only USEGD‐tolerant individuals may continue to participate in the screening program, whereas intolerant individuals may drop out. This discrepancy leads to risk disparities within the target population despite the intention of cancer screening programs to provide equal coverage for all individuals. For patients who found USEGD intolerable, it is essential to provide clear guidance and support, including the option of sedation for subsequent EGD in the context of GC screening, to prevent discontinuation of participation. In settings where recovery logistics or staffing limitations make standard sedation difficult, the flexible use of alternative agents—such as pethidine hydrochloride or newer agents like remimazolam, an ultrashort‐acting benzodiazepine—may serve as viable options to enhance tolerability. Although adequate examination time is essential for diagnostic quality [9, 24], longer procedures may increase patient discomfort. However, in this study, examination time was not associated with severe distress. This may be because subjective discomfort is more influenced by procedural quality, momentary sensations such as gag reflex, and individual sensitivity than by duration itself. Attention should also be paid to appropriate pretreatment and procedural techniques in transnasal endoscopy. Several studies suggest that the tolerability of unsedated transnasal endoscopy is influenced by proper nasal pretreatment, appropriate scope selection, and sufficient staff training. Key strategies include the use of topical lidocaine with vasoconstrictors, careful navigation through the nasal cavity, patient positioning, and supportive voice coaching [25, 26, 27]. In addition, a randomized controlled trial demonstrated that nasal breathing offers better tolerance and operability than oral breathing during transnasal endoscopy [28]. These measures should also be standardized and monitored in GC screening programs utilizing endoscopy.

High participation and continuity are essential for reducing cancer mortality. Modification of behavior through behavioral science and multidisciplinary approaches has been explored to enhance participation rates in cancer screening [29, 30]. The Health Belief Model, the classic framework in health behavior science, highlights perceived barriers—such as pain and stress—as key deterrents [31, 32]. In this study, 10.3% of participants reported being unable to continue with USEGD‐based screening, suggesting that procedural discomfort may pose a significant barrier. Although these individuals were a minority, their needs remain unaddressed, and the potential role of sedation warrants further investigation. In risk communication, the actual risk of USEGD should be clearly conveyed. Although sedation carries a small risk, pursuing zero risk may hinder meaningful practice. Participants have the right to choose between sedative and unsedated EGD after being fully informed, but this right is not always guaranteed in GC screening using endoscopy. Recent European guidelines stress the importance of shared decision‐making [13], which may not yet be sufficiently implemented in Japan.

This study has several limitations. First, the survey was conducted immediately after the procedure, which may have led to response bias, as some participants might have withheld negative feedback out of consideration for the physician. Future studies should consider using patient‐reported outcomes in more neutral settings. Second, despite the availability of ultrathin scopes, more than 20% of procedures were conducted using the transoral approach, but changes in insertion route and scope type were not recorded, potentially introducing bias in tolerance assessment. Nevertheless, such procedural variation reflects real‐world practice, where decisions are made based on anatomy, patient preference, and physician judgment in the absence of standardized regulations. Third, the number of first‐time EGD participants was substantially smaller than planned (134 vs. 400), reducing statistical power and the precision of intolerance estimates (e.g., 26.9% [95% CI: 18.9–36.4]). Although model assumptions were met, the small number of intolerant cases (*n* = 28) may limit the robustness and generalizability of subgroup analyses.

USEGD is the standard procedure for GC screening in Japan and was widely accepted by participants in this survey; however, some individuals found it intolerable, even with the ultrathin endoscope. Supportive measures and targeted interventions are necessary to address these participants’ needs.

## Conflicts of Interest

The authors declare no conflicts of interest.

## Ethics Statement

**Approval of the research protocol by an Institutional Review Board**: This study was approved by the institutional review board of the Showa University Hospital (M3475).

## Consent

The first item of the questionnaire sought consent for participation, and only those who provided consent participated in the questionnaire survey.

## Clinical Trial Registration

N/A.

## Supporting information



Questionnaire form used for on‐site survey in this study.

## Data Availability

N/A
