# Supplementary material for: When Endoscopic Sedation is Not an Option: Insights From a Multicenter on‐site Survey on Tolerance for Japanese Gastric Cancer Screening
Source: DEN Open. 2025 Aug 11;6(1):e70179. doi: 10.1002/deo2.70179 (PMC12338493; doi:10.1002/deo2.70179)
Supplement: Supplementary file 1 — Questionnaire form used for on‐site survey in this study. [file DEO2-6-e70179-s001.docx]

| **Questionnaire survey to investigate the tolerability of esophagogastroduodenoscopy** |
| --- |
| Esophagogastroduodenoscopy (EGD) during cancer screening is performed while the patient is conscious with consideration for safety; however, variability in an individual’s experience of discomfort has been observed during this procedure. Until now, little attention has been given to how patients undergoing screening perceive EGD when performed while conscious." We are therefore conducting a study using a survey regarding EGD to investigate this matter. Personal information will not be identified, and participation or non-participation will not affect a patient’s future care. Your cooperation would be greatly appreciated.  Do you consent to participate in this survey?  □ Yes  □ No  Age ( ) years old. Sex (Man, Woman) |
| 1. How many times have you undergone EGD?  □ First time　□ 2–5 times　□ 6 times or more |
| 2. Have you ever received sedated EGD?  □ I have undergone the examination while asleep.  □ I have not undergone the examination while asleep.  □ This is my first examination.  □ Unsure |
| Please mark the closest number to your feelings with a ○.  (3) How did you feel about today's EGD examination? 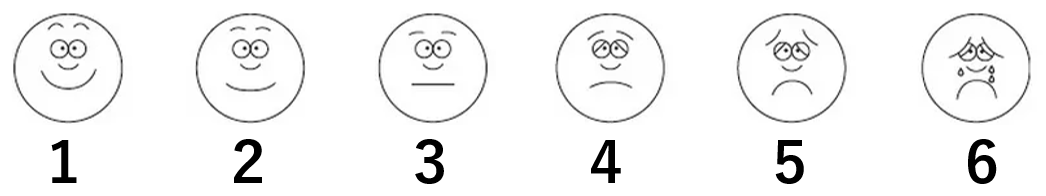 |
| 4. Do you think that you could undergo unsedated EGD again?  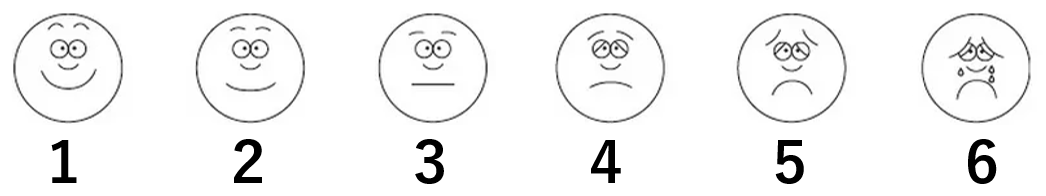 |
| 5. What was your impression of the examination time?  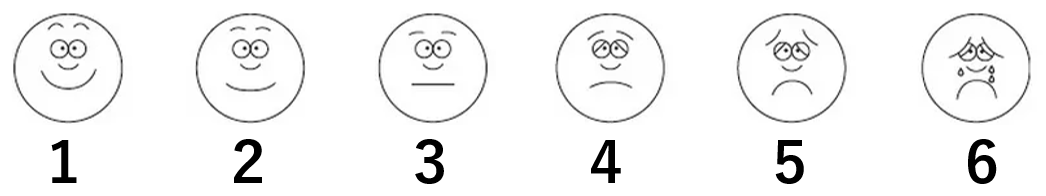 |
| 6. How did you feel after EGD?  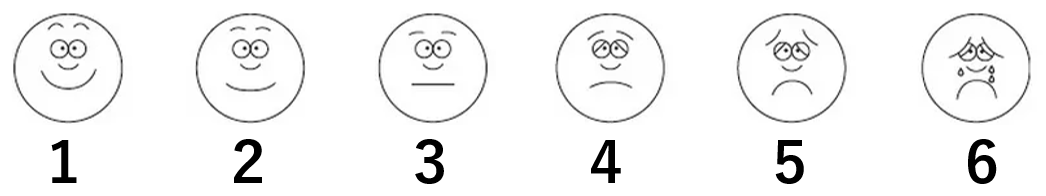 |
| 7. What do you think about the sedation during EGD?  □ Not necessary  □ Would prefer if possible  □ Absolutely necessary |
| 8. Considering the reported adverse events from endoscopic examinations with sedation, as per the 6th National Survey Report on Complications Related to digestive Endoscopy:  Frequency of events:  Hypotension, and respiratory depression: 0.0013% (1 in 80,000)  Death: 0.000024% (1 in 4,166,667)  Please indicate your preference based on these risks:  □ As the risk of adverse events is not zero, I prefer to undergo unsedated EGD.  □ The risk of adverse events is low, so I prefer to undergo sedated EGD while sedated.  □ Other ( ) |
| 9. Considering the limitations associated with undergoing EGD with sedation, including a longer stay at the medical facility (approximately 1 h) and the inability to drive or engage in precise activities on the day of the examination, please indicate your preference:  □ I prefer to undergo esophagogastroduodenoscopy while conscious, taking into account the above limitations.  □ Despite the limitations, I prefer to undergo esophagogastroduodenoscopy while sedated.  □ Other ( ) |
| 10. How do you feel about continuing to undergo esophagogastroduodenoscopy while conscious every few years?  □ I would be comfortable undergoing the procedure while conscious every few years.  □ It would be difficult to continue undergoing the procedure while conscious every few years (I would prefer sedation for the next session). |
| *Medical Institution Section  * Insertion Route  □ Transnasal thin endoscope □ transoral thin endoscope □ transoral regular endoscope  * Biopsy □ Yes □ No  * Examination Time (rounded to seconds)  □ 5 min or less □ 6 mins □ 7 min □ 8–9 min □ 10 min or more |
